# How to Differentiate Hemianesthesia from Left Tactile Neglect: A Preliminary Case Report

**DOI:** 10.3233/BEN-2012-110225

**Published:** 2013

**Authors:** Marco Pitteri, Annalena Venneri, Francesca Meneghello, Konstantinos Priftis

**Affiliations:** ^1^Laboratory of NeuropsychologyIRCCS San Camillo HospitalVenezia-LidoItaly; ^2^Department of NeuroscienceUniversity of Sheffieldand Sheffield Teaching Hospital Foundation NHS TrustSheffieldUK; ^3^Department of General PsychologyUniversity of PadovaPadovaItaly

**Keywords:** Tactile neglect, hemianesthesia, spatial attention, neurological examination, lesion mapping

## Abstract

When assessing for the presence of hemianesthesia, the examiner touches the body of the patients, and requests that they report verbally the location of the delivered tactile stimulus. Contralesional omissions of single tactile stimuli, however, might be due to either primary somatosensory deficits or to spatial attention impairment (i.e., neglect). In this preliminary study, we tested whether clinical assessment can be improved to differentiate between these two types of deficit by modifying the assessment procedure. K.L., a patient with left unilateral neglect, was asked to detect tactile stimuli delivered in two conditions: spatial attention distributed either to his left or to his right hand, and spatial attention focused only on his left hand. Note that K.L. did not receive double simultaneous tactile stimuli. In the distributed spatial attention condition, K.L. omitted most of the single tactile stimuli delivered to his left hand. In the focused attention condition, K.L. was asked to focus his spatial attention only on his left hand. Under this latter condition, his performance increased dramatically, suggesting that his omissions were not due to hemianesthesia, but rather reflected left tactile neglect. In line with the neuropsychological findings, voxel based analysis of his grey and white matter damage confirmed significant loss in areas associated with left-sided neglect, but sparing of the primary somatosensory cortex. This result suggests that standard somatosensory assessment and differential diagnosis between hemianesthesia and tactile neglect may be more accurate when neuropsychology-based procedures are incorporated in the standard neurological examination.

## 1. Introduction

Up to 65% of stroke patients show contralesional somatosensory loss (i.e., hemianesthesia), which can affect their functional outcome [1]. To test for the presence of hemianesthesia, in the standard neurological assessment procedures, typically the examiner touches both hemibodies of the patient, and asks the patient to report verbally the presence, location, and/or intensity of the delivered tactile stimulus. Contralesional omissions of tactile stimuli, however, can be due to either somatosensory or attention deficits. For instance, patients with left unilateral neglect (LUN) following right-hemisphere damage fail to report, orient to, or verbally describe stimuli in the left side of space [2]. LUN is normally due to the patients' failure to orient automatically their spatial attention towards the contralesional side of space [3]. LUN has been observed for each sensory modality (visual, auditory, tactile, etc.). Patients with LUN are able, however, to orient voluntarily their spatial attention focus towards the contralesional side of space when receiving an explicit cue [4].

One of the major diagnostic challenges in the assessment of patients with right hemisphere lesions is ascertaining whether omissions of contralesional stimuli reflect a primary sensory disorder or are the expression of LUN. If patients with right hemisphere damage fail to detect single tactile stimuli delivered on the left limb,

ISSN 0953-4180/13/$27.50 © 2013 – IOS Press and the authors. All rights reserved this often leads to a diagnosis of left hemianesthesia ignoring the possibility that failure to report contralesional stimuli might be the expression of LUN. Indeed, performance of some of these patients might improve substantially when their left hand is positioned within the right side of space [5,6], suggesting that impairments in detecting contralesional stimuli can be due not only to primary somatosensory deficits, but also to LUN. Improvements cannot be attributed to changes in primary somatosensory deficits, because the innervation of the left limb remains the same independently of its position in space (i.e., left or right hemispace). On the contrary, improvements of this kind can only be explained by a change in the orientation of spatial attention, since changing the position of the left limb by moving it away from the neglected side of space (i.e., left) into the intact one (i.e., right) alters spatial attention and consequent processing of the limb. By delivering tactile stimuli to the left limb when positioned either in the left side of space or in the right side of space, the examiner would be able to differentiate between a left sensory deficit due to hemianesthesia and one due to tactile LUN. Moving of the left limb, however, is not always possible or practical because patients can be affected by spastic hemiparesis, pain, or proprioceptive deficits that prevent them from moving towards and/or maintaining their left limb in the right side of space. A possible alternative to overcome this problem might be to ask patients with LUN to orient voluntarily their spatial attention to the left limb during assessment.

We tested this hypothesis in the present preliminary study by asking a patient with LUN (K.L.) and apparent left hemianesthesia to detect, while blindfolded, single tactile stimuli in two conditions: a "divided spatial tactile attention" condition and a "left-oriented spatial tactile attention" condition. The divided spatial tactile attention condition was similar to that included in the standard neurological examination for left hemianesthesia. That is, we delivered sequentially (not simultaneously as it is the case in the examination of tactile extinction) single tactile stimuli either to his left or to his right hand randomly, to make him distribute voluntarily his spatial tactile attention to both hands. In the left-oriented spatial tactile attention condition, K.L. was informed that single tactile stimuli would be delivered exclusively to his left hand. This verbal cue allowed him to focus voluntarily his spatial tactile attention to his left hand only. We hypothesized that if the detection of single tactile stimuli delivered to his left hand was influenced by spatial tactile attention deficits, his stimulus detection rate would be higher in the left-oriented condition than in the distributed condition. In contrast, if K.L.'s detection of left tactile stimuli was not altered by spatial attention cueing, then his left tactile stimulus omissions would reflect the presence of hemianesthesia.

## 2. Methods

### *2.1. Case history*

K.L., a 54-year-old, right-handed man, with 10 years of education, suffered a right hemisphere hemorrhagic stroke in April 2010. The lesion was localized in the territory of the right medial cerebral artery. He had negative neurological and psychiatric history. During hospitalization, K.L. received neuropharmacological treatment and motor therapy for his left hemiparesis. At the time of testing, K.L. was partially able to walk for short distances by himself with the help of a tripod, but he mainly used a wheelchair for long distance mobility. He gave his informed consent to take part in this study, which was conducted accordingly to the principles of the Declaration of Helsinki (revision 2008).

### *2.2. Neuropsychological assessment*

Patient K.L. underwent formal neuropsychological evaluation in June 2010. He was alert and cooperative. K.L. was oriented to time, space, and personal information. His social, behavioral, and emotional control remained normal. General cognitive abilities were preserved and he achieved a score of 25/30 on the Mini Mental State Examination [7]. He had no memory deficits (as tested by digit span [8] and verbal paired-associates learning [9]). His performance on the verbal judgment task [10] was borderline but still within the normal range and his non-verbal reasoning (Raven's Colored Progressive Matrices [11]) was preserved. K.L. showed, however, behavioral signs of LUN including spontaneous head and gaze deviation towards the ipsilesional side of space (i.e., right). His performance on the Behavioral Inattention Test [12] and on the Bells test [13] confirmed the presence of LUN for the visual modality. K.L. was able, however, to explore haptically his left hemibody (Fluff test [14]). K.L.'s neuropsychological data are shown in Table 1.

### *2.3. Procedure*

During examination, K.L. was blindfolded and sat in front of a table. Both his left and his right arms were

Table 1
K.L.'s scores on neuropsychological tests

| Test | K.L.'s score | Cut-off |
|---|---|---|
| MMSE (Folstein et al., 1979) | 25/30 | 24 |
| Digit span (Orsini et al., 1987) | 4/4 | 0/4 |
| Verbal paired-associates learning (Novelli et al. 1986) | 4/4 | 0/4 |
| Verbal judgments (Spinnler and Tognoni, 1987) | 1/4 | 0/4 |
| Raven's Coloured Progressive Matrices (Raven, 1947) | 19.8/36 | 18.96/36 |
| Behavioural Inattention Test (Wilson et al., 1987) | | |
| Conventional part | 122/146* | 129/146 |
| Behavioural part | 61/81* | 67/81 |
| Bells test (Gauthier et al., 1989) | 28/34* | > 3 omissions |
| Fluff test (Cocchini et al., 2001) | 14/15 | > 2 left-sided omissions |

*Score below the cut-off.

placed parallel on the table, in a comfortable position. The examiner checked that K.L. kept his head straight for the duration of the examination. We assessed K.L.'s ability to detect single tactile stimuli, by delivering a touch with a swab on the fleshy part of his fingertips, in two conditions: "divided spatial tactile attention" (e.g., "I am going to touch either your left or your right hand") and "left-oriented spatial tactile attention" (e.g., "I am going to touch only your left hand"). Note that K.L. did not receive double simultaneous tactile stimuli. Each finger was touched three times (15 touches for each hand). Tactile stimuli were delivered randomly among the fingers in variable inter-stimulus intervals.

## 3. Results

### 3.1. Behavioral testing

In the divided attention condition, K.L. showed an apparently severe left tactile hemianesthesia (correct detections of single tactile stimuli on his left hand: 1/15; correct detections of single tactile stimuli on his right hand: 15/15). His performance, however, improved dramatically in the left-oriented condition (correct detections: 13/15), $\chi^2(1) = 19.29, p < 0.001$, suggesting that his apparently severe left tactile hemianesthesia was not due to a primary somatosensory disorder, but reflected tactile LUN. Note that K.L.'s responses were always executed immediately following each touch, showing that he was not guessing.

### 3.2. Structural MRI acquisition, pre-processing, analysis, and results

To verify whether the behavioral data were supported by structural sparing of the primary somatosensory cortex, a morphometric assessment of K.L. was carried out by comparing his grey and white matter values extracted from his 3D brain MRI with those of age-, education-, and gender-matched controls. Three dimensional T1-weighted MRI images of K.L. were acquired on a 1.5 T MRI scanner. Voxel dimensions were $0.937 \times 0.937 \times 1.6$ mm$^3$. The field of view was 240 mm with a matrix size of $256 \times 256 \times 124$. A number of pre-processing steps were followed to isolate the grey matter (GM) and white matter (WM) from the 3D T1-weighted structural scan before performing the statistical analysis using SPM8 (Wellcome Trust Centre for Neuroimaging, UCL, London, UK). To correct for global differences in brain shape, structural images were warped to standard stereotactic space and segmented to extract GM, WM, and cerebral spinal fluid. The GM and WM segments were then modulated to correct for changes in volume, induced by nonlinear normalization, and were smoothed using a Gaussian filter set at 8 mm to reduce possible error from between-subject variability in local anatomy and to render the data more normally distributed. Smoothed GM and WM segments were entered into the analyses. Anatomical regions were identified using the Talairach Daemon Client (http://www.talairach.org/), following conversion of the Montreal Neurological Institute coordinates extracted from the SPM analyses into Talairach coordinates using the Matlab function mni2tal (http://imaging.mrc-cbu.cam.ac.uk/downloads/MNI2tal/mni2tal.m). Height threshold was set at $p < 0.001$ (uncorrected) with an extent threshold of at least 50 voxels. Only clusters surviving family-wise error correction were considered significant.

Two voxel-based independent t-test comparisons were carried out, between K.L.'s GM and WM segments and those of a group of 10 age-, education-, and gender-matched healthy controls (mean age 50.8, *SD* 9.32, mean education 12.8, *SD* 4.96), who were scanned with the same MRI protocol used for K.L. Sig-

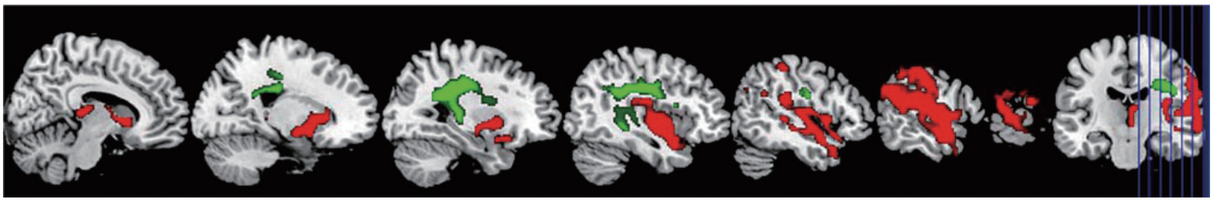

Fig. 1. Note for readers of the printed version: grey matter differences are shown in dark grey, whereas white matter differences are shown in light grey. Grey and white matter differences between K.L. and the group of 10 neurologically healthy controls. Grey matter differences are shown in red; white matter differences are shown in green.

nificant differences between the GM of the patient and that of the controls were found in the region of the right temporal-parietal junction, in the right thalamus, and in the brainstem (see Fig. 1). Similarly, the analysis of the WM segments showed WM loss in parietal and frontal areas of the right hemisphere (Fig. 1). No involvement of the right primary somatosensory cortex was detected by these comparisons, but significant differences were found in the secondary somatosensory cortex (i.e., the inferior part of the postcentral gyrus, BA 2).

## 4. Discussion

K.L.'s detection of tactile stimuli delivered to his left hand improved dramatically when his spatial attention was cued and focused on his left hand. The differential level of left tactile stimuli detection under the two assessment conditions suggests that K.L.'s apparent left hemianesthesia was not due to a primary somatosensory deficit, but rather reflected tactile LUN. This finding suggests that orienting of spatial attention during assessment of tactile perception might be a useful technique for differentiating between a primary somatosensory deficit (i.e., hemianesthesia) and one originating from tactile LUN. The evidence from this single case study suggests that the standard neurological examination should be improved by including both mixed blocks of single tactile stimuli (i.e., single stimuli -not simultaneous- delivered either to the right or to the left hand) and pure blocks of single tactile stimuli (i.e., single stimuli delivered only to the contralesional hand). Our behavioral findings were supported by the evidence from the morphometric study which showed extensive damage of structures in the region of the right temporal-parietal junction, a region associated with spatial attention and damage of which has been extensively shown to result in LUN [15]. No significant difference between K.L. and controls was found in the primary somatosensory cortex (BA 1), although significant differences were detected in the secondary somatosensory cortex (BA 2) in the postcentral gyrus, and in the region of the right parietal operculum, a region which plays an important role in tactile spatial attention [16]. The involvement of this cortical area in K.L.'s brain damage is consistent with the observed behavioral deficits and the successful modulation of detection of tactile stimuli by overt orienting of spatial attention.

The results of our preliminary study suggest that routine neurological examination and differential diagnosis of somatosensory deficits may reach a higher level of accuracy and be more useful for assessment and rehabilitation planning, when neuropsychology principles (e.g., spatial attention orienting) and short additional procedures (e.g., stimulating each hemibody in a separate session) are added to the standard neurological examination. To the best of our knowledge this approach (i.e., using also a pure block with only left-sided tactile stimuli) is not normally performed by clinicians and it is not described in clinical neurology textbooks. An extended group study, however, including both brain-damaged patients and neurologically healthy controls is required for adding internal and external validity to our preliminary behavioral and anatomical findings. The present case study is only the first step in that direction.

## Acknowledgments

We would like to thank K.L. and all controls for their time and effort. We are grateful to Luca Ghezzo, Mariano Trevisan, and dr. Luciano Foscolo for their assistance with the acquisition of MRI scans.
